# The association between ATM variants and risk of breast cancer: a systematic review and meta-analysis

**DOI:** 10.1186/s12885-020-07749-6

**Published:** 2021-01-05

**Authors:** Masoumeh Moslemi, Yousef Moradi, Hojat Dehghanbanadaki, Hamed Afkhami, Mansoor Khaledi, Najmeh Sedighimehr, Javad Fathi, Ehsan Sohrabi

**Affiliations:** 1 Department of Medical Genetics and Molecular Biology, Faculty of Medicine, Iran University of Medical Sciences (IUMS), Tehran, Iran; 2 Department of Epidemiology, School of Public Health, Iran University of Medical Sciences, Tehran, Iran; 3 Students Scientific Research Center, Tehran University of Medical Sciences, Tehran, Iran; 4 Department of Microbiology, Faculty of Medicine, Shahed University, Tehran, Iran; 5 Department of Physiotherapy School of Rehabilitation, Shiraz University of Medical Sciences, Shiraz, Iran; 6 Department of Bacteriology and Virology, School of Medicine, Shiraz University of Medical Sciences, Shiraz, Iran

**Keywords:** Breast cancer, ATM, Variant, Systematic review, Meta-analysis

## Abstract

**Background:**

Ataxia telangiectasia-mutated (ATM) gene contributes to repair damaged DNA and to regulate cell cycle; therefore, ATM variants seem to increase breast cancer risk; however, the results are controversial. So we conducted a systematic review and meta-analysis to clarify the pooled association between various ATM variants and the risk of breast cancer.

**Methods:**

The relevant studies were searched through Scopus, Web of Science, PubMed and Cochrane. Stratified and subgroup analyses were performed to explore heterogeneity between studies and assess effects of study quality. The pooled estimates logarithm with standard error logarithm of odds ratio and relative risk with confidence interval were calculated.

**Results:**

This study revealed that there is association between ATM variants and the risk of breast cancer; according to the seven adjusted case-control studies, OR of this association was estimated as 1.67 (95%CI: 0.73–3.82), according to nine unadjusted case-control studies, the crude OR was 2.27 (95% CI: 1.17–4.40) and according to two cohorts, the RR was estimated as 1.68 (95% CI: 1.17–2.40).

**Conclusions:**

The ATM variants are associated with an increased risk of breast cancer that ATM V2424G mutation is detected as the most predisposing factor while ATM D1853V, L546V, and S707P variants have the least predictive ability.

## Background

Breast Cancer is the most common cancer in women and causes the highest mortality rate in developed and developing countries [1]. Annually, 1.67 million women become infected with this type of cancer and have 522,000 mortality rate each year [2]. According to the World Health Organization (WHO) report, 1 out of 9 women in the world suffers from this type of cancer during their life. The Clinical manifestations of breast cancer are very different in patients and these various manifestations largely depend on the type of genetic mutation. Accurate diagnosis of cancer based on the type of mutation is very helpful for deciding on the treatment and follow up of patients [3].

During the last decade, BRCA1 and BRCA2 mutations (BRCA1/2) have been screened for hereditary breast cancer and results showed that mutations in BRCA1/2 account for 5% of hereditary breast cancer [1, 4]. Besides, a recent large population study showed that other pathogenic gene variants, including ataxia telangiectasia-mutated (ATM) variants, were frequently detected among breast cancer women [5, 6]. The ATM gene is located on 11q22–23 chromosome and consists of 66 exons that encode a 350 kDa protein kinase enzyme [7, 8]. This gene plays an important role in regulating the cell cycle and repairing the DNA damage induced by ionizing radiation. Exposure to ionizing radiation, even in very low doses, can trigger a break down significant amount of cellular inactive homodimer ATM to active monomers [9, 10]. The activated ATM phosphorylates a number of its downstream targets such as p53, chek2 and BRCA1 which stops the cell cycle, repairs DNA or apoptosis, so a mutation in either of these genes causes insufficient cellular repair and ultimately increases the incidence of cancer [9, 11].

Mutations in the ATM gene caused Ataxia Telangiectasia (A-T), an autosomal recessive syndrome that patients have symptoms such as ionizing radiation sensitivity, cerebellar neurodegeneration immunodeficiency and markedly increased risk of cancers like breast cancer [12–14]. The incidence of breast cancer among female relatives of A-T families was increased [15–18]. Also, Epidemiologic studies have estimated that obligate heterozygous carriers of an ATM mutation have a 2–5-fold increased risk of breast cancer [13, 19, 20]. In addition, it is reported that breast cancer patients with mutated ATM variants who undergo radiotherapy developed their second tumor sooner than the group with no treatment of radiation and no ATM mutations [21].

Since the detection of the ATM gene in 1995, several studies have been conducted on the association of mutations of this gene and the risk of breast cancer which had contradictory results and to our knowledge, no systematic study was conducted to examine the association between different variants of ATM and breast cancer. Therefore, the current study aims to clarify the pooled relationship between ATM variants and breast cancer.

## Main text

### Methods

All methods used in this meta-analysis were according to the Preferred Reporting Items for Systematic Reviews and Meta-Analyses statement (PRISMA) [22]. The protocol of this study had been registered in the International Prospective Register of Systematic Reviews (PROSPERO), under the registration number of CRD42018114394.

### Search terms and complex search syntax

To evaluate the relationship between breast cancer and Ataxia Telangiectasia Mutated Proteins, four English databases including Scopus, Web of Science (EMBASE), PubMed, and Cochrane were browsed up to 20 October 2018. In current study “breast carcinoma”, “breast tumor”, “breast neoplasm”, “breast neoplasms”, “breast cancer”,” breast cancers”, “breast tumors”, “ataxia telangiectasia mutated”, “ataxia telangiectasia mutated proteins”, “ATM”,” mutation”, “mutations”, “variant”, and “variants” keywords were searched in the mentioned databases. Because of avoiding missing any papers, the reference list of primary articles was screened manually. The primary search results were reviewed, and some articles were eliminated after reviewing their title and abstract. Inclusion and exclusion criteria were set by 2 researchers separately (YM, MM).

### Eligibility criteria

Articles were selected using the following criteria: (1) assessment of the association between ATM variants and breast cancer risk; (2) studies with full text articles; (3) case–control and cohort studies; (4) sufficient data for estimating an odds ratio (OR) with 95% confidence interval (CI).

### Screening and data extraction

Full texts of each article were sensibly assessed by two independent reviewers (ES and MM) and any probable disagreements with these reviewers were resolved by consultation with another author (YM) to settle the argument, and a concluding deduction was applied. The following data were collected from each study: first author’s name, publication date, type of study, country, study population, age, sample size, type of variant, measurement of association and controlled variables.

### Quality assessment and risk of Bias

The qualities of all studies were assessed by Modified Newcastle-Ottawa Scale for Case Control studies [23]. This checklist was completed by two researchers (ES and MM). The quality of studies was judged based on such aspects as selection, comparability and outcome. Scores ranged from 0 stars (worst case) to 9 stars (best case). Studies with a score of 0–4 were categorized as low quality, 5–7 as moderate, and more than 7 as high qualities.

### Statistical analysis

Association between the ATM and breast cancer risk was analyzed by pooling odds ratio (ORs) and risk ratio (RR) with 95% confidence interval (CIs) using STATA metan module. DerSimonian and Laird method were used to compute the pooled estimate of odds ratio (OR) and risk ratio (RR) with a confidence interval (CI 95%) using random models [24]. Because the test for heterogeneity was statistically significant in some analyses, the random effects models were used to estimate OR and RR. In the fixed effects model approach, we used two popular methods, which included the inverse variance-weighted average method and the weighted sum of the z-scores method. Although previous studies have shown that the two methods perform similarly, their characteristics and their relationship have not been thoroughly investigated. Therefore, in present meta-analysis we used the inverse variance-weighted model [25, 26]. In this study, w Cochran’s Q test and I square statistic was used to evaluate statistical heterogeneity between studies [27]. In addition, a meta-regression and subgroup analysis were performed to assess the source of heterogeneity between studies. Moreover, publication bias was assessed by funnel plot and Egger test [28, 29]. Statistical analysis was performed using STATA 14.0 (Stata Corp, College Station, TX, USA) and statistical significance was set at *p* < 0.05.

## Results

### Study characteristics

**Fig. 1:**
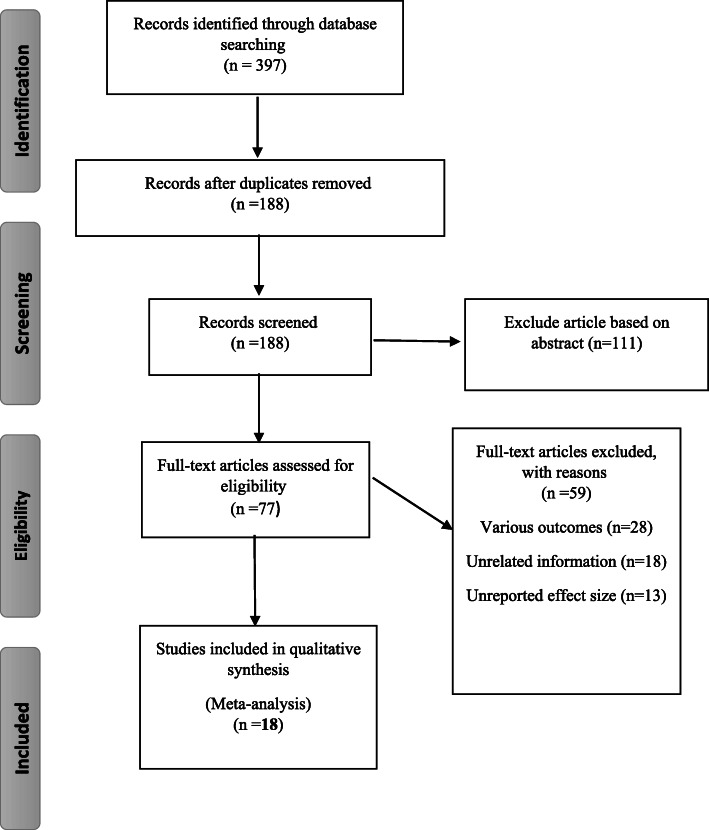
PRISMA chart outlining the literature search

The first step of search in electronic databases yielded 397 publications. In the final step, after removing the duplicates, reviewing by title, abstract and full text and considering the inclusion and exclusion criteria, 18 studies were selected for the meta-analysis of the pooled association between ATM and the risk of breast cancer (Fig. 1). The characteristics of each study included in the current meta-analysis are reported in Tables 1 and 2.

**Table 1:** The main characteristics of cohort and Population based cohort studies of ATM variants on risk of Breast cancer

Authors (years) (country)	Study population	Age	Sample size	Type of variant	Measurement of association	Controlled variables	NOS Score
Nic Waddell. et al. (15) (2006) (South Australia)	South Australia and Royal Melbourne Hospita	All age	782	7271 T > G (V2424G)	hazard ratio of 6.1 (95% CI: 1.2–30.8)	multiple-case non-BRCA1 and non- BRCA2 breast cancer families	7
Sarah Louise Dombernowsky. et al. (5) (2008) (Denmark) [11]	Danish general population		10,324	Ser49Cys	Age Adjusted HR 95% CI 1.0 (0.5–2.2)	Heterozygotes versus Alcohol consumption Smoking habits Reproductive history	7
					Multifactorial Adjusted HR 95% CI 0.8 (0.3–2.0)		
				Ser707Pro	Age Adjusted HR 95% CI 0.60 (0.2–1.5)		
					Multifactorial Adjusted HR 95% CI 0.6 (0.2–1.6)

**Table 2:** The main characteristics of Case Controls studies of ATM variants on risk of Breast cancer

Authors (years) (country)	Study population	Age	Sample size	Type of variant	Measurement of association	Controlled variables	NOS Score
Thomas A. Buchholz;et al.(2004,USA)	Patients with breast carcinoma between 1997 to 2000, and Control participants did not have a personal history of malignant disease	All age	1031Total 940(case )91 control	Ser49Cys	odds ratio [OR] breast carcinoma: 5.52 (1.89–16.12) bilateral breast carcinoma: 10.31 (2.12–50.13)	Age; Ethnicity; History of second malignancy; Family history of breast carcinoma; Stage and Type of surgery	7 7
Melissa C Southey. et al. (2016, Australia) [30]]	Participants were drawn from studies participating in The Breast Cancer Association Consortium (BCAC)	All age	Total 848,355 (Case 42,671) Control42164)	c.7271 T > G p.Val2424Gly	OR: 11.6 (1.50 to 89.9)	-.	666 6
Philip Bretsky. et al. (2003, California)	Participants were recruited between 1993 and 1996	45–75 years	Total (854) (Case 428) Control 426)	L546V	OR: 3.35 (1.27–8.84)	Age, Ethnicity, family history of breast cancer	6 6
J.L Bernstein et al. (2006, the San Francisco, California; Ontario, Canada, Melbourne, Sydney and Australia) [9]	Diagnosed between 1995 and 1998 were identified	All age	Total (5011) Case (3743) Control (1268)	c.7271 T > G Heterozygous	OR: 8.6 (3.9–18.9)	Age	7 7
				c.1066–6 T > G Heterozygous	OR: 0.4 (0.2–1.0)		
Hong Ding. et al. (2010, China)	There were six studies of population-based design and five studies of hospital-based design	All age	Total (13788) Case (8831) Control (4957)	C.1066-6 T < G	OR: 0.87 (0.55–1.37)	–	77 7
Teresa Tapia. et al. (Chile 2007,)	Sample was obtained from a blood bank, Santiago, Chile.	All age	Total (294) Case (94) Control (200)	c. 2572 T > C, (p. F858L)	0.53 OR: 0.07–3.55))	–	7 7 7 8 8 8 7 7 6
				c.1744 T > C, (p. F582L)	(20.3–0.92) 4.32		
				T > C2119 (p.S707P)	(1.63–0.04) 0.26		
				IVS24-9delT	(3.05–0.99) 1.74		
				IVS38–8 T > C	(2.05–0.45) 0.96		
				c.5557 G > A, p.D1853N	(4.77–1.33) 2.52		
				IVS38–8 T > C, c.5557G > A	(4.64–0.51) 1.53		
				IVS24-9delT and c.5557G > A ((p.D1853N	(8.40–1.87) 3.97		
Brennan Decker. et al. (2017,UK)	A population-based study of breast cancer (BC) in the region of East Anglia (UK)	All age	Total (18575) Case (13087) Control (5488)	c.7390 T > C (p.Cys2464Arg, rs55801750,	OR: 0.37 (0.19–0.73)	Gene length, with approximately 60 variants per kilobase of coding sequence	7
Deborah Thompson; et al.(19) (2005,Australia) [20]	case-control-family study which recruited subjects between 1992 and 1999 from metropolitan Melbourne and Sydney, Australia	To age 70	Total (1153) Case (775) Control (378)	c.1066-6 T > G (IVS10-6 T > G)	HR: 3.4 (0.80–11.0)	–	7
			Total (457) Case (84) Control (373)	c.4258C > T (p.Leu1420Phe)	HR: 0.8 (0.3 to 1.8)		
Csilla I. Szabo. et al. (2004, Austria)	All families were originally referred to clinics for medical care and were later selected for research purposes for including at least two cases of invasive breast cancer in first- or second-degree relatives.	before age 60 years	Total (1715) Case (1172): 961 FH (non-BRCA1/2) cases and 211 FH (BRCA1/2) cases) Control (543)	IVS10–6TG	OR: 1.60 (0.48–5.35)	Sample that did not carry a pathogenic mutation of the BRCA1 or BRCA2genes	8
Denise L. Stredrick. et al. (2006, USA and Poland)	The study of ATM was initiated in a nested breast cancer case–control study within the U.S. Radiologic Technologist (USRT) cohort study, Blood samples were collected between 1999 and 2004 And The second study is a case–control investigation of breast cancer conducted in Poland from 2000 to 2003	All age	(USRT) 1048 control Case 861 (Poland) 3037 cases and 3639 control	(c.146C > G, p.S49C)	Poland, OR: 1.88 (1.17–3.02) USRT, OR: 1.60 (0.88–2.90) Combined, OR: 1.69 (1.19–2.40)	Study group (USRTor Poland) and Age (below 50, 50^59, 60^69, 70+ years)	8
				S707P	Poland, OR: 1.25 (0.80–1.94) USRT, OR: 0.47 (0.23–0.93) Combined, OR: 0.92 (0.65–1.32)		
				F858L	Poland, OR: 1.12 (0.67–1.86) USRT, OR: 2.03 (1.05–3.90) Combined, OR: 1.44 (0.98–2.11)		
				P1526P	Poland, OR: 1.02 (0.86–1.22) USRT, OR: 0.75 (0.49–1.13) Combined, OR: 0.93 (0.79–1.09)		
Jonine L. Bernstein (2017, USA)	All women in the WECARE Study were interviewed and complete medical treatment history information was collected	All age	Total (2107) Control (1399) )708(case	c.1899-55 T > G	Relative risk (RR): 0.5 (0.3–0.8)	- unilateral breast cancer (UBC)	8
				c.3161C > G	0.5 (0.3–0.9)		
				c.6348-54 T > C	0.2 (0.1–0.8)		
				c.5558A > T	0.2 (0.1–0.6)		
T. Stankovic, et al. (1998, British)	Lymphoblastic cell lines (LCLs) from 78 patients in 68 A-T families	All age	Total (603) Controls (202) Case (401)	c.7271 T > G	(Relative risk: 12.7)	–	7
Patricio González-Hormazábal. (2008, South American (Chili))	Chilean families were selected from the files of the Metropolitan Santiago National Health Service, National Cancer Society	All age	Total (326) Case (126) Control (200)	IVS24-9delT, IVS38–8 T > C, 5557G > A	T/(−T), T/T, G/A OR: 1.31 [0.63–2.66]	BRCA1/2 negative breast cancer cases and controls.	6
					T(/−T), T/C, G/A OR: 3.19 [1.16–8.89]		
				IVS24-9delT, 5557G > A	T/(−T), G/A OR: 1.74 [0.96–3.16]		
				IVS38–8 T > C, 5557G > A	T/T, G/A OR: 1.31 [0.63–2.66]		
					T/C, G/A OR: 1.31 [0.63–2.66]		
				IVS24-9delT, IVS38–8 T > C	T/(−T), T/T OR: 1.31 [0.63–2.66]		
					T/(−T), T/C OR: 3.19 [1.16–8.89]		
				G > A 5557	(G/A) OR: 1.74 [0.96–3.16]		
					(A allele) OR: 1.67 [0.94–2.92]		
				IVS38–8 T > C	(T/C) OR: 3.09 [1.11–8.59]		
					(C allele) OR: 3.00 [1.09–8.21]		
				IVS24-9delT	(T/−T) OR: 1.74 [0.96–3.16]		
					(−T) allele OR: 1.67 [0.94–2.92]		
Johanna Tommiska. et al. (2006, Helsinki)	The familial breast cancer patients have been collected bya systematic interview for family history at the Helsinki University Central Hospital	All age	Total (1592) Case (884) Control (708)	ATMex39 D1853N	Unselected breast cancer patients OR: 0.89 [0.72–1.09]	–	7
					Familial breast cancer patients OR: 0.89 [0.73–1.10]		
					Index with one affected first degree relative OR: 0.85 [0.67–1.07]		
					Three or more affected in the family OR: 0.98 [0.74–1.30]		
					Unselected breast cancer patients, Bilateral OR: 0.60 [0.32–1.11]		
					Familial breast cancer patients, Bilateral OR: 0.94 [0.59–1.49]		
				ATMivs38(−8)T > C	Unselected breast cancer patients OR: 1.06 [0.69–1.64]		
					Familial breast cancer patients OR: 1.32 [0.87–2.02]		
					Index with one affected first degree relative OR: 1.47 [0.93–2.31]		
					Three or more affected in the family OR: 1.08 [0.60–1.95]		
					Unselected breast cancer patients, Bilateral OR: 1.43 [0.49–4.15]		
					Familial breast cancer patients, Bilateral OR: 0.96 [0.40–2.32]		
Amanda B Spurdle. et al. (2002, Australian)	Cases were women with a diagnosis of a first primary breast cancer identified and Controls were women without breast cancer selected from the electoral roll Using stratified random sampling, frequency-matched for age.	59	Total (3835) cases (1300) controls (600)	C2119	OR: 1.08 (0.59–1.97)	age, country of birth, state, education, marital status, number of live births, height, weight, age at menarche, oral contraceptive use, and reported family	8
				G3161	OR: 1.30 (0.85–1.98)		
Kirsi Määttä, et al. (Swede 2016,)	Hereditary breast and /or ovarian cancer (HBOC) families were used for whole exome sequencing.	All age	Total (1118) Case (129) Control (989)	c.2572T4C p.(F858L)))	0.75 (0.10–5.92)	BRCA1/2 founder variant-negative HBOC	7 7
				c.3161C4G p.(P1054R)))	0.54 (0.07–4.13)		
				c.5558A4T p.(D1853V)))	1.54 (0.18–13.19)		

### Association between ATM and breast Cancer in case control studies

#### Adjusted case-control studies

**Fig. 2:**
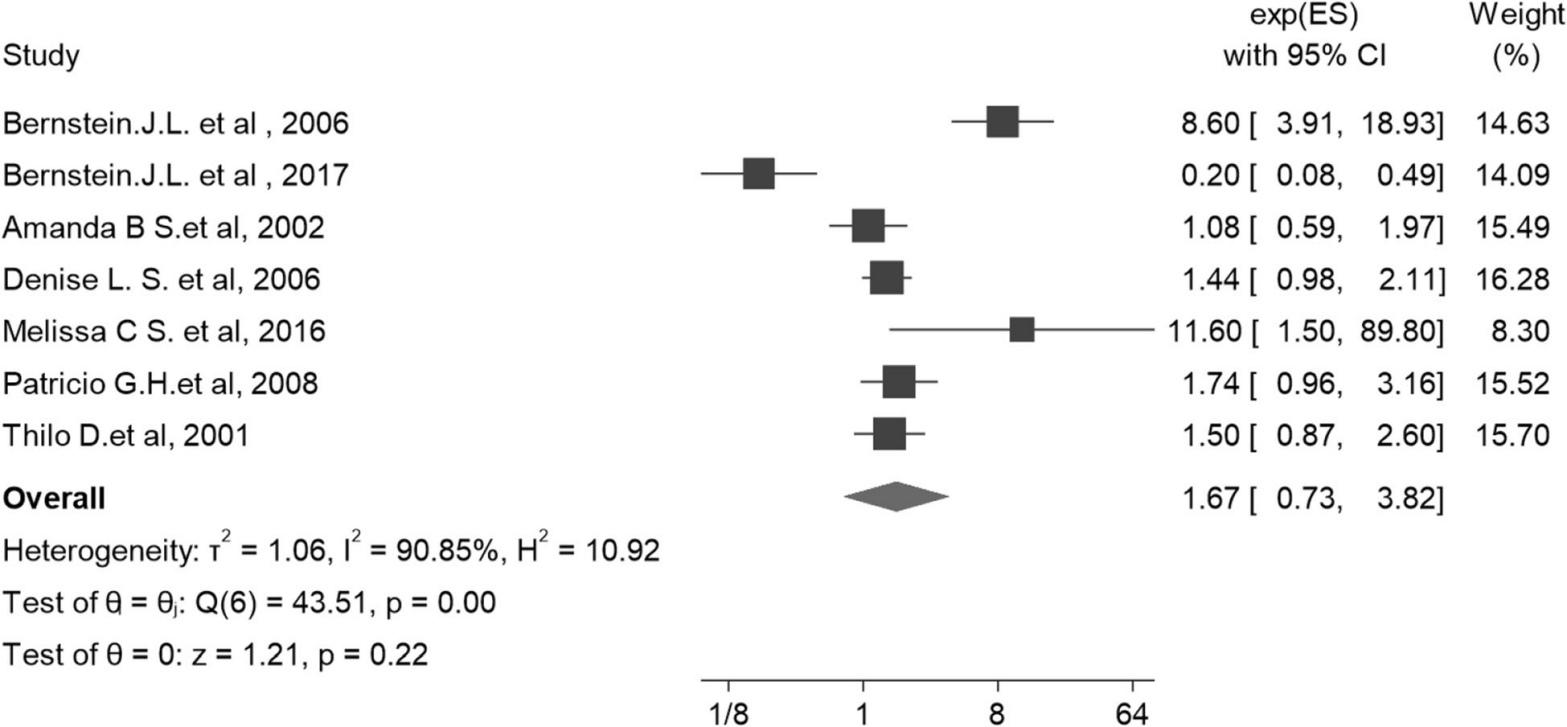
The forest plot of Association between ATM and Breast Cancer in Adjusted Case Control Studies (Adjusted Odds Ratio)

The pooled estimate odds ratio after synthesis results of 7 Adjusted case control studies shows that the association between ATM and risk of breast cancer was 1.67 (95% CI: 0.73–3.82; I square: 90.85%; P_I square_: 0.0001). The range of OR between these studies was 0.2 to 11.60 (Fig. 2). The results of Egger’s test showed there isn’t a publication bias in the association between ATM and risk of breast cancer (Coefficient = 2.08; *P* = 0.193; % 95 CI: − 0.56 – 3.16).

The results of subgroup analysis show that the highest association between ATM variants and the risk of breast cancer belongs to the Asian population with an odds ratio of 4.21 (95%CI: 0.78–22.88; I square: 89.5%; P_I square_: 0.0001) while the lowest odds ratio was among the European population that was equal to 1.24 (95% CI: 0.94–1.64; I square: 18.5%; P_I square_: 0.297). This suggests that ATM variants have a greater impact on the risk of breast cancer in the Asian population than in the European and American populations (Table 3).

**Table 3:** Summary odds ratio (OR) or relative risk (RR) Estimates [95% confidence intervals (CIs)] for observational studies conducted on The association between ATM variants and risk of breast cancer

Subgroup	Number OR in primary studies	Summery effect size (95% CI)	Between studies			Between subgroups	
			I^2^	P _heterogeneity_	z	Q	P _heterogeneity_
Adjusted Case Control studies							
Heterozygosity variants							
Hetro	13	1.98 (1.22–3.19)	80%	0.0001	2.78	5.03	0.0001
Hemo	4	1.63 (1.20–2.22)	0.0%	0.929	2.12		
Hetro/Hemo	7	1.22 (1.01–1.46)	0.0%	0.433	3.14		
Continent							
Asia	2	4.21 (0.78–22.88)	89.5%	0.0001	1.67	4.03	0.0001
America	3	1.55 (1.19–2.01)	60.4%	0.001	3.22		
European	2	1.24 (0.94–1.64)	18.5%	0.297	1.49		
Type							
c.7271 T > G	2	8.94 (4.28–18.67)	0.0%	0.789	5.83		
D1853V	2	0.43 (0.09–2.07)	78.6%	0.031	1.05		
S707P	3	1.17 (0.73–1.87)	49.2%	0.140	0.66	4.03	0.0001
F858L	2	1.43 (1.02–2.00)	0.0%	0.943	2.10		
IVS38–8 T > C, c.5557G > A	2	1.90 (0.80–4.48)	48.9%	0.162	1.46		
IVS38–8 T > C	2	3.04 (1.48–6.25)	0.0%	0.963	3.04		
IVS24-9delT	2	1.70 (1.13–2.57)	0.0%	0.922	2.54		
5557G > A	3	1.33 (0.89–1.99)	54.7%	0.110	1.39		
IVS24-9delT, IVS38–8 T > C,	2	1.90 (0.80–4.48)	48.9%	0.162	1.46		
5557G > A	2	1.90 (0.80–4.48)	48.9%	0.162	1.46		
IVS24-9delT, IVS38–8 T > C							
Unadjusted Case Control studies							
Heterozygosity variants							
Hetro	28	1.31 (1.05–1.64)	76.3%	0.0001	2.35	3.89	0.001
Hetro/Hemo	3	3.43 (0.99–11.84)	90.9%	0.0001	1.95		
Continent							
Asia	3	5.37 (1.66–17.48)	86.5%	0.0001	2.79		
America	2	1.58 (1.07–2.33)	67.8%	0.0001	2.32	3.89	0.0001
European	4	1.08 (0.85–1.38)	74.8%	0.0001	0.63		
Type							
c.1066–6 T > G	8	1.69 (0.91–3.14)	78.1%	0.0001	1.67		
Ser49Cys	3	9.24 (4.78–17.87)	0.0%	0.468	6.61		
7271 T > G (V2424G)	2	27.97 (5.01–35.07)	62%	0.105	3.80	3.89	0.0001
IVS38–8 T > C	6	1.22 (0.98–1.51)	0.0%	0.874	1.78		
D1853N	6	1.22 (0.98–1.51)	58.9%	0.032	0.67		

The results based on homo/heterozygosity status of variants show that heterozygous variants had increased the risk of breast cancer with a factor of 1.98 (95% CI 1.22–3.19; I square: 80%; P_I square_: 0.0001). Also there is an association between homozygous variants and the risk of breast cancer with an odds ratio (OR) of 1.63 (95% CI: 1.20–2.22; I square: 0.00%; P_I square_: 0.929). Meanwhile, the lowest OR belonged to homo or heterozygous which was equal to 1.22 (95% CI: 1.01–1.46; I square: 0.00%; P_I square_: 0.433). This statistical analysis indicates that heterozygous ATM variants are the most associated with breast cancer incidence (Table 3).

In addition, results of subgroup analysis based on type of variants show that the V2424G variant (c.7271 T > G) is the most associated with breast cancer incidence (OR: 8.94; %95 CI: 4.28–18.67; I square = 0.00%; P_I square_ = 0.789) and D1853V has the least impact on breast cancer incidence (OR: 0.43; 95% CI: 0.09–2.07; 0.031; I square: 78.6%; P_I square_: 0.789) (Table 3).

#### Unadjusted case-control studies

**Fig. 3:**
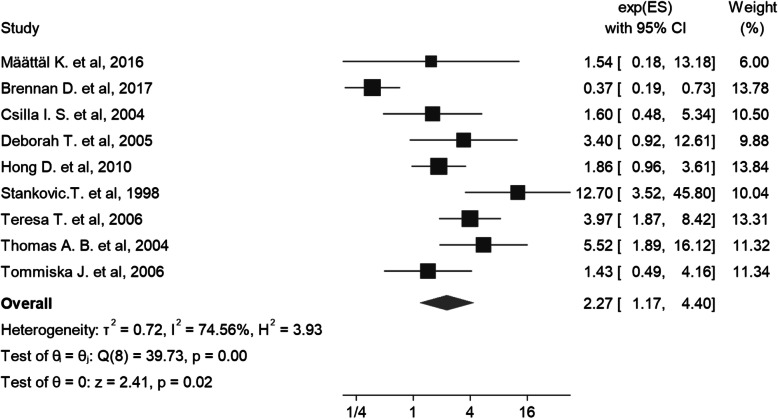
The forest plot of Association between ATM and Breast Cancer in unadjusted Case Control Studies (Crude Odds Ratio)

From the 9 unadjusted case control studies, the association between ATM variants and the breast cancer risk ranged between 0.37 and 12.70. The pooled estimate of crude odds ratio between ATM and risk of breast cancer was 2.27 (95% CI: 1.17–4.40; I square: 74.56%; P_I square_: 0.0001) (Fig. 3). The results of Egger’s test showed no publication bias in the association between ATM and risk of breast cancer in unadjusted case control studies (Coefficient: 0.398; P: 0.193; % 95 CI: − 0.21 – 1.00).

Subgroup analysis based on continent shows that ATM variants have a greater impact on breast cancer incidence in the Asian population than in the European and American populations (Table 3). In addition, the heterozygote ATM variants increased the risk of breast cancer 1.31 times (95% CI: 1.05–1.64; I Square: 83.0%; P_I square_: 0.0001) (Table 3).

Also, the evaluation of the effect of different variants of ATM Gene on the incidence of breast cancer shows that c.7271 T > G has the highest association with an OR of 27.97 (95% CI: 5.01–35.07; I Square: 62.0%; P_I square_: 0.105) and L546V has the lowest association with an OR of 0.37 (95% CI 0.19–0.73; 0.032; I Square:58.9; P_I square_: 0.105) (Table 3).

### Association between ATM and breast Cancer in cohort studies

#### Cohort studies

The results of two cohort studies showed a risk ratio (RR) range of 0.6 to 6.10. The pooled risk ratio between ATM variants and risk of breast cancer was 1.68 (95% CI: 1.17–2.40; 0.032; I square: 62.1%; P_I square_: 0.105).

## Discussion

ATM gene has been recognized in recent years as a low penetrance breast cancer gene, which is a research goal for many studies [31]. Based on the mentioned keywords 18 articles were evaluated and different variants of this gene in three continents of Asia, America and Africa were analyzed. Qualitative assessment of articles was performed by NOS criteria and the results showed that all articles are of medium to high quality, so they met the criteria for entering meta-analysis. The results of the current study indicate that ATM missense variants increase the incidence of breast cancer and people who carry these variants have an increased risk of developing breast cancer.

In the study by Easton DF et al. [32] in 2015, it is reported that relatives of A-T patients encounter breast cancer with a relative risk of 2.8 (90%CI: 2.2–3.7; *P* < 0.0001) [32]. Another study by van Os NJH et al. [33] in 2016 demonstrated that all women with pathogenic ATM variants developed breast cancer with a relative risk of 3.04 (90%CI: 2.06–4.48; *P* < 0.0001). Another meta-analysis of 19 studies by Marabelli M et al. [34] in 2016 reported that the women who were the carriers of ATM variants have a breast cancer relative risk of 6.02 (90%CI: 4.58–7.42) by the age of 50 years old and 32.83 (90%CI: 24.55–40.43) by the age of 80 years old [34]. As the same as our findings, all these studies emphasized that there is a strong association between ATM variants and the risk of breast cancer development.

Of the 29 different variants examined in the current study, V2424G (c.7271 T > G) missense variant had the highest association with breast cancer incidence in different subgroups of adjusted case control, non-adjusted case control and cohort studies. Although this association varied across the three subgroups analyses, all revealed that V2424G missense variant was strongly accompanied by an increased risk of breast cancer. Bernstein JL et al. [9] in 2006 reported that women who were carriers with V2424G missense variant had developed breast cancer by the age of 70 years old with a cumulative risk of 52% (95% CI = 28–80%; *P* < 0.0001). Another study by Goldgar DE et al. [35] in 2011 analyzed 15 different A-T families and revealed that pathogenic V2424G variant accompanied with increased risk of breast cancer with a relative risk of 8 (95% CI: 2.3–27.4; *P* = 0.0005. In addition, another study by Southey MC et al. [30] in 2016 reported that V2424G missense variant was detected in 12 of 42,671 patients suffered from invasive breast cancer and one of 42,164 normal control population that these statistics results in an OR of 11.0 (95% CI:1.42–85.7; *p* = 0.0012). Also, Mitui M et al. [36] evaluated the clinical consequences of ATM gene alterations using stable transfection and among the 12 missense variants examined, ATM V2424G variant was one of the variants which were associated with an increased risk of cancer.

The meta-analysis on the adjusted case-control studies revealed that ATM D1853V missense variant has the least association with an increased risk of breast cancer. Gao LB et al. [37] in 2010 revealed that there is significantly no relationship between the D1853V missense variant and the risk of breast cancer development [37] that this finding is congruent with our results.

Another objective of our study was to investigate the association of ATM variants with breast cancer in different regions. Although the incidence of breast cancer is higher in the Americas and Europe than in the Asian continent [38, 39], it is interesting to note that the association between ATM variants and breast cancer is greater in Asian countries than in American and European countries in all three sub-groups analyses that is probably due to racial differences, environmental conditions, patterns of life and the effects of other genes or specific haplotype combinations in that region [39].

The overall risk heterogeneity in our study was 67.4, which could be due to the way we measured, sample-sizes designs, inclusion criteria, family history and BRCA1/2 mutation status and could distort the interpretation of our results, so we performed a sub-group analysis based on variant type, region and heterozygosity to illustrate this heterogeneous factor and thus reduced the heterogeneity up to zero.

### Limitation

Since the primary studies used in this meta-analysis did not provide sufficient information about the exact age of the patients, the type of breast cancer, its stage and grade, the hereditary or sporadic type and unilateral or bilateral breast cancer, further subgroup analysis was not possible. Furthermore, there are few cohort studies in this area, and we need more studies.

## Conclusion

This meta-analysis shows that the pathogenic ATM variants are associated with an increased risk of breast cancer. Accordingly, ATM variants, including V2424G have the highest risk of breast cancer incidence while ATM D1853V, L546V, and S707P variants have the least impact on breast cancer incidence.

## Data Availability

All data generated or analyzed during this study are included in this published article.

## Abbreviations

CI: Confidence Interval
RR: Risk Ratio or Relative Risk
OR: Odds Ratio
BMI: Body Weight Index
CINAHL: Cumulative Index to Nursing and Allied Health Literature
EMBASE: Excerpta Medica dataBASE
PRISMA: Preferred Reporting Items for Systematic Reviews and Meta-Analyses statement
NOS: Modified Newcastle-Ottawa Scale
ATM: Ataxia telangiectasia-mutated
STROBE: Strengthening the Reporting of Observationally Studies in Epidemiology
PROSPERO: International Prospective Register of Systematic Reviews
